# Computer-assisted contralateral side comparison of the ankle joint using flat panel technology

**DOI:** 10.1007/s11548-021-02329-w

**Published:** 2021-04-20

**Authors:** Sarina Thomas, Lisa Kausch, Holger Kunze, Maxim Privalov, André Klein, Jan El Barbari, Celia Martin Vicario, Jochen Franke, Klaus Maier-Hein

**Affiliations:** 1 Division of Medical Image Computing, German Cancer Research Center, Heidelberg, Germany; 2 Medical faculty Heidelberg, Universität Heidelberg, Heidelberg, Germany; 3 Imaging and Therapy Systems Division, Siemens Healthineers, Erlangen, Germany; 4 Division of Medical Imaging and Navigation in Trauma & Orthopaedic Surgery, BG Unfallklinik Ludwigshafen, Ludwigshafen, Germany

**Keywords:** Plane estimation, Segmentation, Computer-assisted surgery

## Abstract

**Purpose:**

Reduction and osteosynthesis of ankle fractures is a challenging surgical procedure when it comes to the verification of the reduction result. Evaluation is conducted using intra-operative imaging of the injured ankle and depends on the expertise of the surgeon. Studies suggest that intra-individual variance of the ankle bone shape and pose is considerably lower than the inter-individual variance. It stands to reason that the information gain from the healthy contralateral side can help to improve the evaluation.

**Method:**

In this paper, an assistance system is proposed that provides a side-to-side view of the two ankle joints for visual comparison and instant evaluation using only one 3D C-arm image. Two convolutional neural networks (CNN) are employed to extract the relevant image regions and pose information of each ankle so that they can be aligned with each other. A first U-Net uses a sliding window to predict the location of each ankle. The standard plane estimation is formulated as segmentation problem so that a second U-Net predicts the three viewing planes for alignment.

**Results:**

Experiments were conducted to assess the accuracy of the individual steps on 218 unilateral ankle datasets as well as the overall performance on 7 bilateral ankle datasets. The experiments on unilateral ankles yield a median position-to-plane error of $0.73\pm 1.36$ mm and a median angular error between 2.98$^\circ$ and 3.71$^\circ$ for the plane normals.

**Conclusion:**

Standard plane estimation via segmentation outperforms direct pose regression. Furthermore, the complete pipeline was evaluated including ankle detection and subsequent plane estimation on bilateral datasets. The proposed pipeline enables a direct contralateral side comparison without additional radiation. This has the potential to ease and improve the intra-operative evaluation for the surgeons in the future and reduce the need for revision surgery.

## Introduction

Fractures of the ankle joint are among the most common fractures of the lower extremity and can be challenging to treat [1]. The syndesmotic compound that stabilizes the adjacent bones in the ankle joint is often damaged in association with a fracture. This may lead to an instability of the joint that must be treated intra-operatively. The most common procedure is an open reduction and internal fixation (ORIF). Reduction refers to the re-alignment of the dislocated bones whereas fixation refers to the attachment of metal implants to fixate the bones. Anatomical reduction is essential and incorrect reduction (malreduction) may cause instability and chondral degeneration leading to premature osteoarthritis [5]. Intra-operative imaging allows monitoring of the reduction result and implant positioning during all phases of an intervention. In clinical routine, either fluoroscopic projections or a 3D volume are acquired by a mobile C-arm to verify the reduction of the tibio-fibular joint region. Different studies [3] showed that fibula malreduction may remain overseen in using fluoroscopy and recommend to use 3D imaging. After the image acquisition, the surgeon adjusts the multi-planar reconstruction (MPR) viewing planes of the reconstructed C-arm volume to the anatomy-specific so-called standard planes. Standard planes are defined by clinicians for different anatomical structures and show anatomical key features. Their diligent adjustment is necessary to achieve a reliable diagnosis. The adjustment of the standard planes must be repeated for each scan and increases the intervention time. Despite visual inspection, verification of the tibio-fibular joint reduction is challenging and based on the individual experience due to the high inter-individual variability of the ankle. Mukhopadhyay et al. [4] suggest to compare the reduction outcome to the unimpaired contralateral ankle due to the significant lower intra-individual variability. Acquiring an additional 3D scan of the unimpaired ankle is not implemented in clinical routine since it increases the applied radiation dose and time.

Computer-assistance has found its way into the modern operating room to assist a great variety of surgical procedures. However, in fibular reduction, the procedure is entirely based on manual visual inspection of the acquired image data. Thus, the question has arisen whether computer-assisted methods can be applied to enable a comparison without the need for an additional full scan. In Thomas et al. [2], the first step towards a computer-assisted contralateral side comparison was proposed. The workflow requires a 3D scan of the impaired ankle together with low-dose 2D projection images of the unimpaired ankle. Besides the algorithmic complexity, the approach requires the additional acquisition of images.

**Fig. 1:**
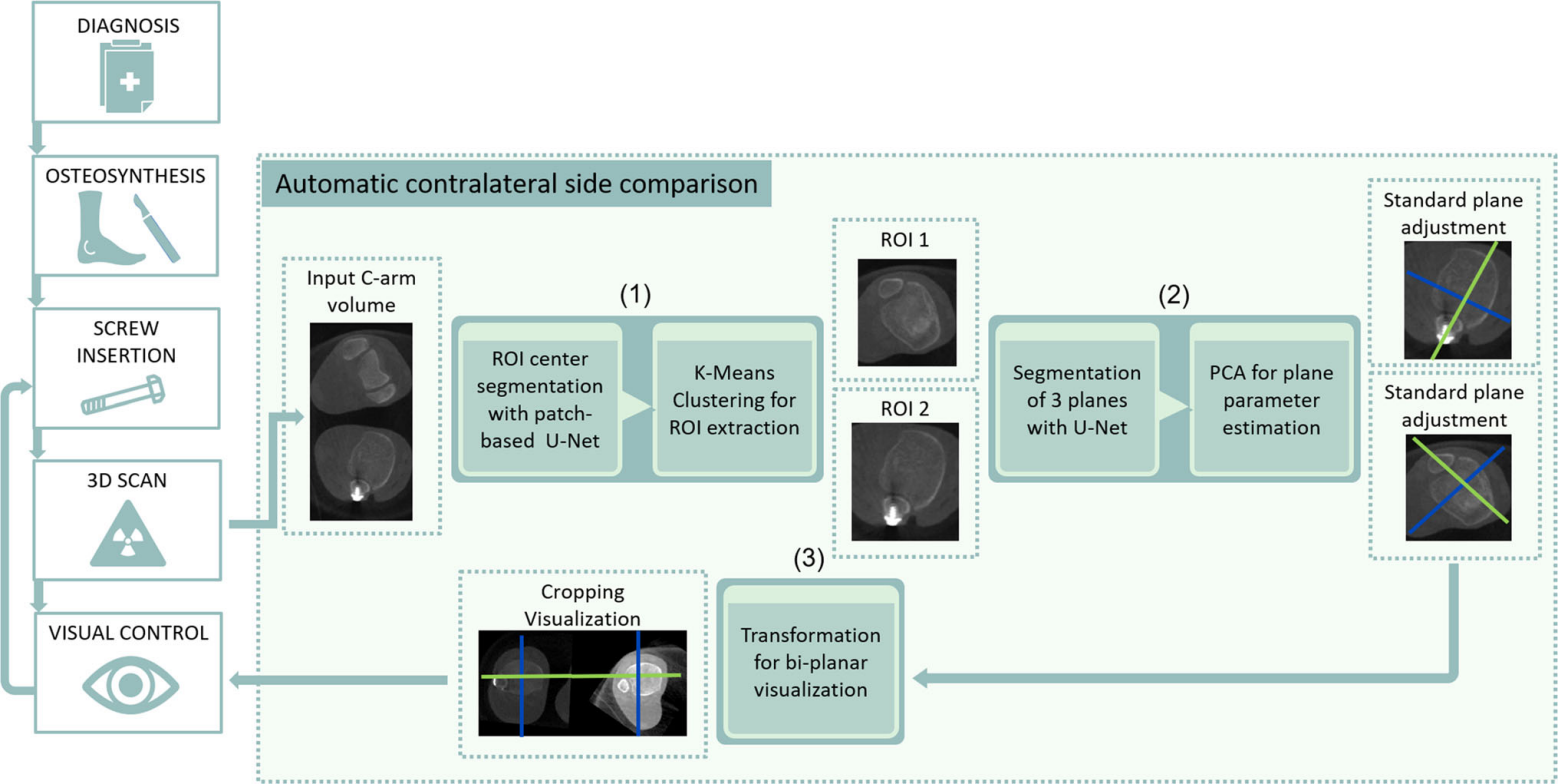
Clinical workflow with the proposed pipeline: After ORIF and image acquisition, (1) both ankles are localized in the 3D image by applying the ROI center segmentation and clustering. (2) On each ROI, the standard plane estimation is performed. (3) The resulting subregions are aligned and visualized

In this work, the first step towards automatic contralateral side comparison using a single C-arm image volume is proposed to extend the clinical workflow (cf. Fig. 1). By computing a side-by-side view, the application allows surgeons to directly compare both ankles on relevant anatomical landmarks. To visualize the ankles in a meaningful way, their relative pose has to be established and aligned. Standard planes can serve as a good pose reference for the anatomy. The main contributions of this work are two-fold: (1) A pipeline for automatically aligning and visualizing symmetric anatomical structures to ease the evaluation is introduced. This paper presents an automatic contralateral side comparison with single volumes and shows that the pipeline can be successfully applied to the ankle joint. (2) The problem of standard plane estimation is solved through an entirely different approach and its feasibility is examined.

## Related work

This section briefly revises the previous work on computer-assisted contralateral side comparison. Since the proposed work focuses on the computation of standard image planes, the section also provides an overview of the state of the art research.

In the literature, only a few examples of contralateral side comparisons are reported to this date. Fürnstrahl et al. [8] presented a pre-operative assistance system for comparison of anatomies with a symmetric counterpart in CT images. The system is targeted at the reconstruction of comminuted fractures of the proximal humerus by mirroring and registration of the unimpaired humerus. In previous work [2], contralateral side comparison is solved by reconstruction of the unimpaired ankle with 3 projection images. A statistical shape model creates a correspondence model of the impaired ankle. The shape model is adapted to bone masks extracted from the projections images by a deep neural network to create the respective unimpaired model. The resulting models can be compared to evaluate pose and shape differences. The complex pipeline was tested on a limited number of ankle 2D and 3D images acquired with an image intensifier. Those images are not normalized and can contain geometrical distortion. More recent developments in flat panel detectors allow a wider field of view (FOV) and better image quality. A flat panel detector can cover both ankles in one C-arm scan. This entails lower radiation dose and time consumption for C-arm movement and image acquisition. To compare the fibula position in the incisura tibio-fibularis of both ankles directly it is important to guide the surgeon to the relevant viewing plane. For that reason, the pose of each ankle must be revealed. The standard plane definition serves as a good standardized representation of the pose. To this date, the standard planes are adjusted manually in the clinical routine but some automatic approaches can be found in the literature.

Brehler et al. [6] calculate the standard planes for the calcaneotalar joint in mobile C-arm images by extracting unique SURF features used for atlas-based registration. The approach is limited to rigid registrations between features calculated from the image and the atlas. In the case of the ankle joint, the positional and rotational relation between fibula and tibia varies and the plane definition depends on both bones. To overcome the limitations, in [9] an approach is presented to use a multi-bone shape model as an atlas that modifies the planes independently. However, a shape model needs to be trained on manual segmentations and errors in shape model segmentation directly affect the adjustment result. Lu et al. [10] detect a 2D standard plane in echocardiography 3D images using a knowledge-based probabilistic model. They reported an overall position error of $3.7 \pm 2.1$ mm and an angular error of $11.3 \pm 3.7^\circ$.

More recent approaches exploit different neural network architectures to solve the plane estimation task. Hou et al. [13] introduced the idea of a regression network that predicts the transformation parameters of a moving image. They aim to predict a good estimate for a subsequent 2D/3D registration. An iterative regression network is proposed by Li et al. [12] to regress standard planes from ultrasound images. Instead of the direct transformation parameters, they predict relative pose updates to be independent of any reference coordinate systems. Their experiments on 72 fetal ultrasound images yielded a positional error of 3.83 mm and 3.80 mm and an angular error of $12.7^{\circ }$ and $12.6^{\circ }$. Most related to the proposed work, Martin Vicario et al. [11] use a CNN to directly regress coordinate system parameters for each of the three viewing planes of the ankle and calcaneotalar joint. They defined the coordinate system for each plane with a 6D representation. They report a median angular error of 6–8$^\circ$ and a positional error of 5 mm.

However, all approaches do not yet fulfill the accuracy requirement of the proposed application since already small plane estimation errors affect the visual comparison. A preliminary survey with clinicians showed that angular errors above 5–10 degrees and more than 1 mm lead to different visual impressions that might affect the evaluation adversely. Furthermore, the regression and registration approaches can not be easily extended to images with more than one instance. In this work, the problem of standard plane estimation is solved differently by defining a segmentation task for all three planes and deriving plane parameters from the resulting masks. The field of medical image segmentation has been dominated by deep learning-based algorithms, in particular different variants of the U-Net architecture [14]. This symmetric encoder-decoder network allows predicting labels for each image voxel. Although extensive research has been undertaken to optimize the architecture, Isensee et al. [7] showed that the original architecture remains state of the art in recent segmentation challenges. It is hypothesized that a segmentation task can further increase the accuracy and lead to more robust plane estimation results.

## Material and Methods

The proposed system is not limited but intended to be used intra-operatively. Hence, some relevant insights on the targeted application of ankle surgery are provided. In the remainder of the section, the different methods and necessary steps towards a contralateral side comparison are described.

### Data

During ankle surgery, a mobile C-arm is used to acquire images of the impaired ankle joint. This device has the great advantage of mobility but on that account holds no standardized information on the device-to-patient orientation. Thus, after tomographic reconstruction, the surgeon needs to adjust the multiplanar reconstruction (MPR) viewing planes of each 3D scan so that relevant areas of the anatomy are visible. Regarding the ankle joint, standard planes are adjusted so that the axial standard plane is orthogonal to the tibial shaft. The sagittal standard plane is orthogonal to the axial plane and builds the angle bisector between the medial contour of the fibula and the tibia at talar dome level approximately 6 mm below the tibial plafond. All planes intersect in the middle of the lowermost tibial plafond. This point is referred to as the intersection point in the remainder of the paper. By scrolling along the axial plane the surgeon can analyze the reduction result at two levels - approximately 10 mm above the tibial plafond to examine the fibula in the incisural notch and 6 mm below to examine the tibia and fibula at the talar dome level. To this date, the surgeon positions only the relevant ankle in the FOV of the C-arm. As a consequence, all available data contains one ankle.

### Workflow

For contralateral side comparison using a single image volume, the following main challenges need to be tackled. The first step towards a comparison is an instance-based detection to localize both ankles in the image. Since only unilateral ankle images are acquired in clinical routine, the entire pipeline needs to be trained on images with one ankle. However, in the final application it also needs to be applicable on bilateral ankle images. So the first task will be to localize and separate both ankles in the volume. After localization, the second task is to compute a pose estimate of each ankle. Once the two poses are established, they can be aligned in a third task so that the viewing planes show the same perspective in a side-by-side manner. This section describes the overall processing pipeline with the underlying methods. Figure 1 shows a typical surgical workflow that is extended by the proposed pipeline. The main components can be separated into 1) Localization 2) Plane estimation and 3) Visualization and are described in details below:

**Fig. 2:**
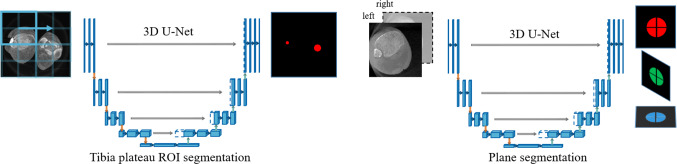
Left: Image patches are inserted into a 3D U-Net and fused afterward to predict a binary mask with a region of interest containing the tibia plateau as foreground. Right: Based on the region of interest extracted by the first U-Net, a second 3D U-Net predicts three masks representing a cylinder for each plane

**Fig. 3:**
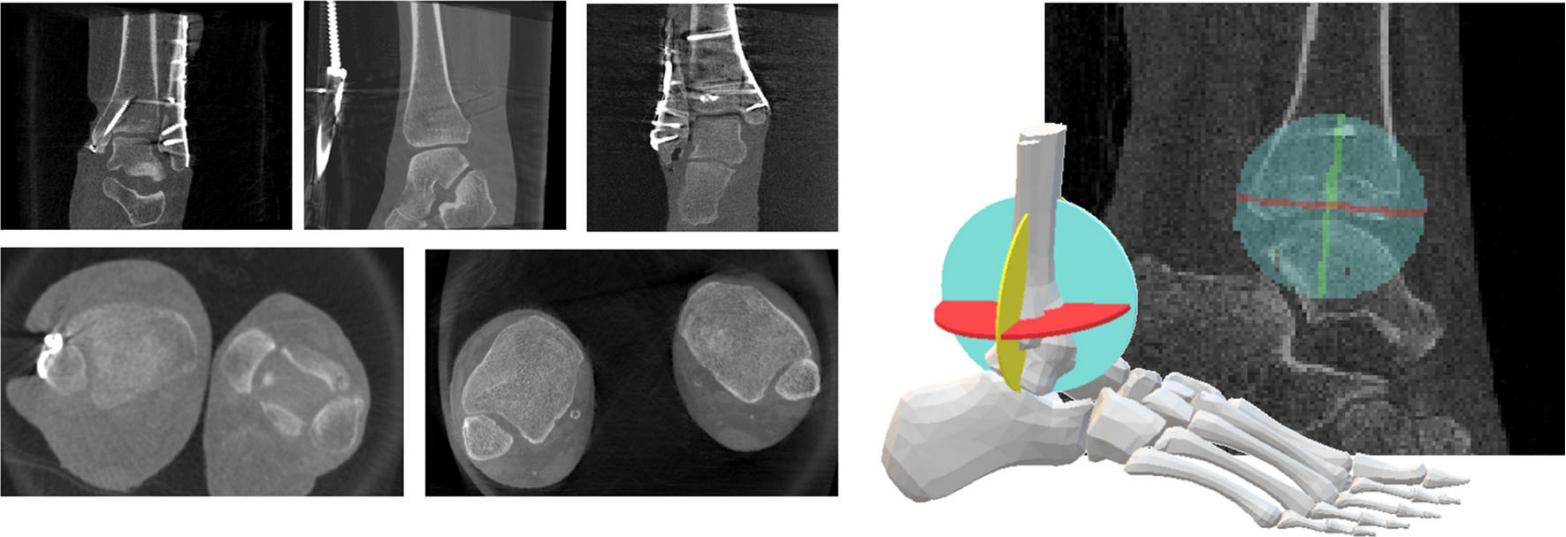
Left: Uni- and bilateral C-arm images of the ankle joint. Right: Standard planes defined as segmentation masks (saggittal, coronal, axial)

**Fig. 4:**
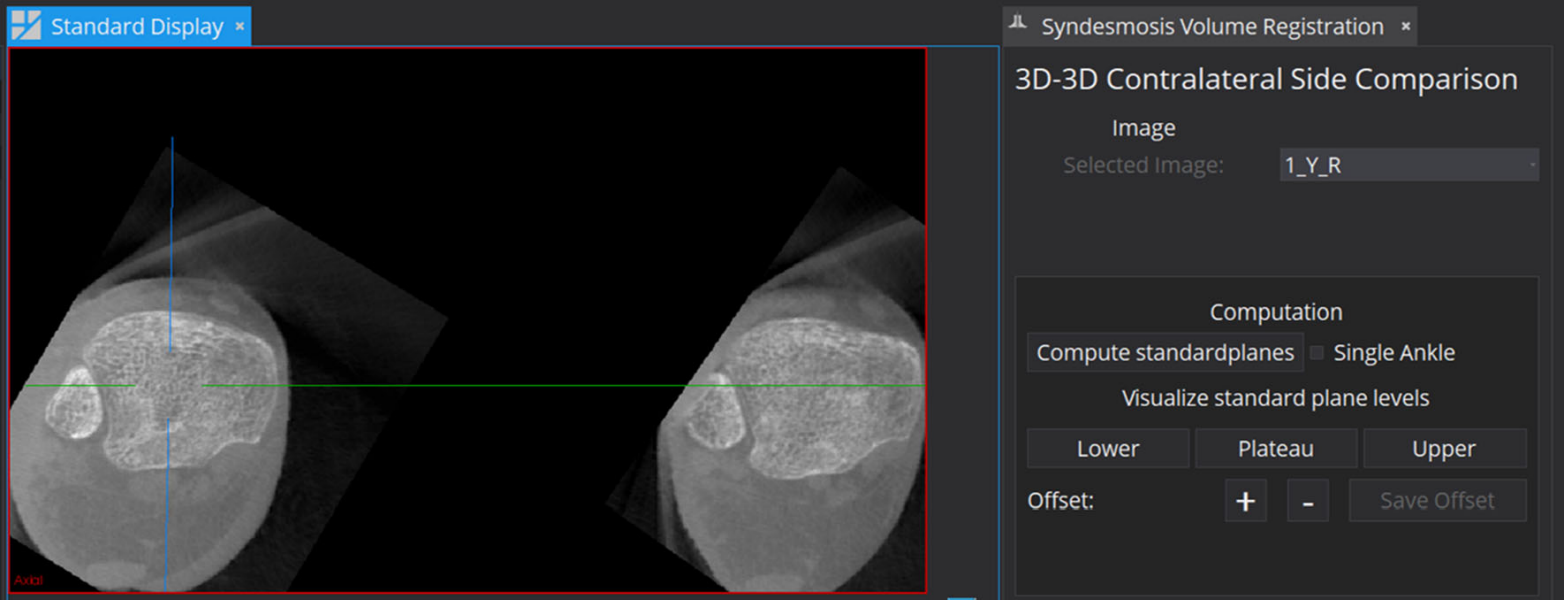
MITK plugin to perform the contralateral side comparison. The standard planes can be visualized on three distinct levels (lower, plateau and upper)

**1) Localization of the tibia plateau:** The two ankles in the image can be arbitrarily placed with respect to the C-arm. The first task is to detect all tibia plateaus in the image to know where relevant subregions are located. The intersection point of the three annotated standard planes can be derived and used as a landmark for the localization. The localization problem is defined as a segmentation problem. Since a single voxel-sized landmark is likely to vanish during image interpolation, the landmark is defined as a sphere with a radius of 5 mm. A standard U-Net based on the original architecture is extended to allow 3D images as input [17]. Given the 3D input images and corresponding segmentations holding discretized sphere masks, the network is trained to predict the location of the sphere in unseen images. The result is a binary mask with all voxels labeled in close proximity to the tibia plateau center. The algorithm is trained on images with one ankle but needs to be applied to images with two ankles. Therefore, not the whole image is presented to the network but smaller patches that only contain a fraction of the ankle. This way the network does not get distracted by images with two ankles. The algorithm iterates over the entire image by extracting overlapping patches (cf. Fig. 2, left). For each voxel, the mean of all predictions is computed. The tibia plateau center must be estimated from the resulting prediction. A simple center of mass computation would fail when two ankles or falsely labeled voxels are present. In contrast, a k-Means clustering on the resulting segmentation assures that the clusters in presence of multiple ankles are separated correctly. K-Means clustering is commonly applied if the number of clusters is known in advance. After averaging overlapping patches, the result is not binary and therefore each predicted average value is applied as a weight for the k-Means algorithm to make the center computation more robust to outliers. After localizing the tibia plateau landmarks, each subregion can be cropped centered around the landmark. This allows the subsequent step to focus on the relevant image content. The larger the patch size is chosen, the more information is fed into the network. On the contrary, large patch sizes pose the risk of containing parts of the second ankle or other exterior structures that may distract the network.

**2) Plane estimation:** Regarding the ankle joint, all three standard planes are pair-wise orthogonal so that its representation can be reduced to a single coordinate matrix. This matrix is composed of a normal for each plane as well as a common intersection point. Instead of defining and regressing the matrix parameters directly, the problem can be also solved through an auxiliary segmentation task. Each plane can be represented by a mask where all image voxels intersecting the plane are labeled (cf. Fig. 3, right). Plane parameters can be derived from this mask representation subsequently. The pose of the viewing plane is solely dependent on the specific anatomy of interest. Hence, the labeled voxels are limited to a circular plane with a radius surrounding the intersection points. To avoid information loss during resizing and interpolation, the circular plane is extended to a cylinder with a certain height. A second 3D U-Net is employed to segment the three resulting cylinders representing the standard planes. The network consists of four output channels, one representing the background and the remaining representing the three axes. Since the cylinders are orthogonal, they intersect and shape a cross (cf. Fig. 2, right). The areas of intersection are omitted to avoid overlapping labels. The segmented cylinders are used to derive plane parameters such as the position and normal. A principal component analysis is applied to the spatial indices of each of the three segmentations separately. The first and second largest eigenvalues account for the cylinder axis. The vector assigned to the third-largest eigenvalue directs towards cylinder height that equals the plane normal. The mean value of all three segmentations approximates the intersection point. Although the cylinder volumes are discontinuous, those regions do neither affect the principal component analysis nor the center point estimation.

**3) Visualization** For contralateral side comparison, the two ankles should be displayed side-by-side to the surgeon. For that purpose, a prototype has been developed as a plugin integrated into the open-source framework Medical Imaging and Interaction Toolkit (MITK) [15]. This framework offers a medical viewer that can be used for loading, analyzing, and viewing of the ankle dataset. With the implemented plugin extension, the ankle dataset can be processed in a single-click manner. The developed plugin (cf. Fig. 4) offers a single and double ankle mode. If one ankle is in the FOV the plugin can be used to display the respective standard plane. The result of the network is fed back into the plugin and used to align all three MPR viewing planes. In the case of two ankles, two sets of standard planes are returned by the network. A subregion around the standard plane center is cropped from the original images for each subset. Both subregions are aligned according to their standard planes. Since the left and right ankle are mirrored, the planes are used to build one left and one right-handed coordinate system. Those systems aligned and mirrored to create the same laterality. Afterward, both ankles are aligned in reference to the intersection point at the lower tibia plateau on the same level side-by-side. At this particular level, the surgeon cannot extract relevant information. Instead, in the clinical workflow, the surgeon will look at the two levels described in Sect. 3.1. The prototype has three operation buttons that allow the user to switch between the lower, plateau and upper level automatically. Even small angle deviations or position changes will cause a different visual plane representation noticeable by the user. According to the clinicians, positional changes of the axial plane are most relevant since they change the level at which the results are examined. Hence, the prototype is equipped with a correction tool that optimizes the axial position in case of position deviations. The correction tool simply uses a ’plus’ and ’minus’ button to translate the position along the axial normal to change the viewing plane if necessary. Internally, the MITK plugin is equipped with a Python-based service that allows 1) transferring the input data and returning results 2) executing a Python script. The service transfers the input data to a script in which the image is processed and inserted into both networks. The Python script consists of a preprocessing part for downsampling the image as well as an interference part in which the networks with the trained weights are executed. Furthermore, the script incorporates postprocessing steps such as the aforementioned k-Means clustering and the principal components analysis. After computing all parameters, the Python script returns the resulting segmentation image and all outputs to the plugin, where the image is cropped and aligned accordingly.

## Experiments and results

**Data acquisition:** For all experiments, Cios Spin ^®^ (Siemens Healthcare GmbH) mobile C-arms were used to acquire 3D images. This mobile 3D C-arm system is equipped with a flat panel detector, offers an isocentric design and $192^\circ$ orbital movement. Each volume is reconstructed from 200 single projection images. The C-arm detector covers an area of $30^2$ cm$^2$ and the reconstruction covers a volume of $16^3$ cm$^3$ and has an isotropic voxel spacing of 0.3 mm with $512^3$ voxels.

The experiments were conducted using datasets from five different sources:

(1) Unilateral ankle data:

- This dataset consists of 66 image volumes. All were acquired in clinical routine after ORIF. The implant occurrence is dependent on the fracture type and ranks from single syndesmotic screws to multiple osteosynthetic plates and screws. All 3D images were acquired at the BG Trauma Center Ludwigshafen after 2018 and anonymized for the retrospective evaluation.
- This dataset consists of 36 image volumes. The volumes were acquired during a surgical training course and cover a wide range of complex osteosynthetic procedures that were performed on cadaveric specimens. This dataset contains a considerable amount of different metal implants and many volumes suffer from large areas of metal artifacts.
- This dataset contains 116 volumes of unimpaired ankles. They were acquired using cadaveric specimens and partly contain external metal objects. No metal objects were present in close proximity to the joint center.

All image volumes have in common that they contain only a single ankle joint.

(2) Bilateral ankle data

- This dataset consists of 4 C-arm image volumes. The cadaveric specimens were scanned before surgical training. All specimens had two unimpaired ankle joints that were covered with a thin plastic layer and fixated tightly with rubber-tape to fit into the FOV. No metal implants were present in the FOV.
- The remaining 3 image volumes were taken during surgical intervention at the BG Trauma Center Ludwigshafen. Both ankle joints were surgically disinfected and fixated to fit into the field of view of the scanner.

For the experiments, standard planes of all datasets were annotated using a standard plane adjustment plugin integrated into the MITK workbench.

**Fig. 5:**
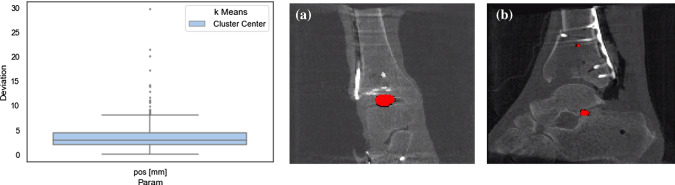
Localization of the tibia plateau: Accuracy evaluated on the unilateral ankle data in a 5-fold cross-validation. The boxplot shows the deviation between the ground truth and the predicted intersection point. **a** Shows the segmentation of an example with a low positional deviation whereas **b** shows the segmentation of an outlier example with the highest positional deviation

**Data processing and augmentation:** All image volumes were downsampled from 512 to 160 voxels and normalized with zero mean and unite variance. Extensive online augmentation was applied to the image volumes using batch generators [18]. A random spatial transform rotates the image along each of the axes in the range of $[-45,45]^\circ$. A random scaling of [0.8, 1.2] accounts for the naturally occurring size differences in humans. Offset augmentation is realized by random cropping of the volumes depending on the respective input size of the network. To enable an invariance towards anatomical laterality, the images are also mirrored along the image axes. Mirroring can only approximate the reality because it assumes that left and right ankles are truly symmetrical. Studies showed that the intra-individual variability is significantly lower than the inter-individual variability [5]. The input data consists of left and right ankles to generalize towards intra-individual variability eventually caused by biomechanical attrition. For network optimization, a cross-entropy and soft dice loss is combined that proved to be suitable for segmentation tasks [7]. The loss is optimized using an ADAM optimizer with an initial learning rate of $3e^{-4}$ [16].

**Table 1:** Plane estimation: Accuracy comparison (median$\pm \sigma$) of the proposed approach of plane estimation via an auxiliary segmentation task to direct CNN regression (results were taken from [11]) on 218 unilateral ankle data. The lower table shows the overall system performance (plane estimation with prior localization) on three clinical cases and four cadaver specimen with bilateral ankle data.

	pos [mm]	pos2Ax [mm]	sag [$^\circ$]	axial [$^\circ$]	cor [$^\circ$]
**Unilateral data**					
Proposed method	$1.77\pm 0.21$	$0.72\pm 0.15$	$3.32\pm 0.32$	$2.98\pm 0.32$	$3.71\pm 0.43$
SOTA [11]	N/A	$6.61\pm 0.40$	$9.18\pm 1.22$	$5.20\pm 0.97$	$7.57\pm 0.77$
**Bilateral data**					
Specimen	$2.09\pm 0.52$	$0.96\pm 0.74$	$3.74\pm 2.52$	$2.80\pm 1.64$	$3.02\pm 3.60$
Clinical	$1.17\pm 0.49$	$0.35\pm 0.20$	$2.60\pm 1.10$	$2.28\pm 1.44$	$2.16\pm 0.87$

**Evaluation metrics:** For evaluation, the 3D Euclidean distance deviation (pos) as well as the shortest point-to-plane distance (pos2Ax) of the axial plane were measured between the ground truth and the predicted intersection point. The latter is more important since it directly refers to the visualized level whereas the in-plane distance is less prominent for the visual perception. Additionally, the angular deviation between the axial (axial), sagittal (sag) and coronal (cor) normal vector and the respective ground truth standard plane was examined.

**Localization of the tibia plateau:** In this experiment, the accuracy of the network for localization of the tibiotalar joint space in C-arm images was evaluated. The U-Net was trained on the unilateral ankle data in a 5-fold manner so that a test set is excluded from the training to estimate the error. The remaining four sets are subdivided in three training and one validation set. The crop size was set to $64^3$ voxels and the patches were sampled randomly from the image. The batch size was set to 16 image patches and the network was trained for 1000 epochs.

**Fig. 6:**
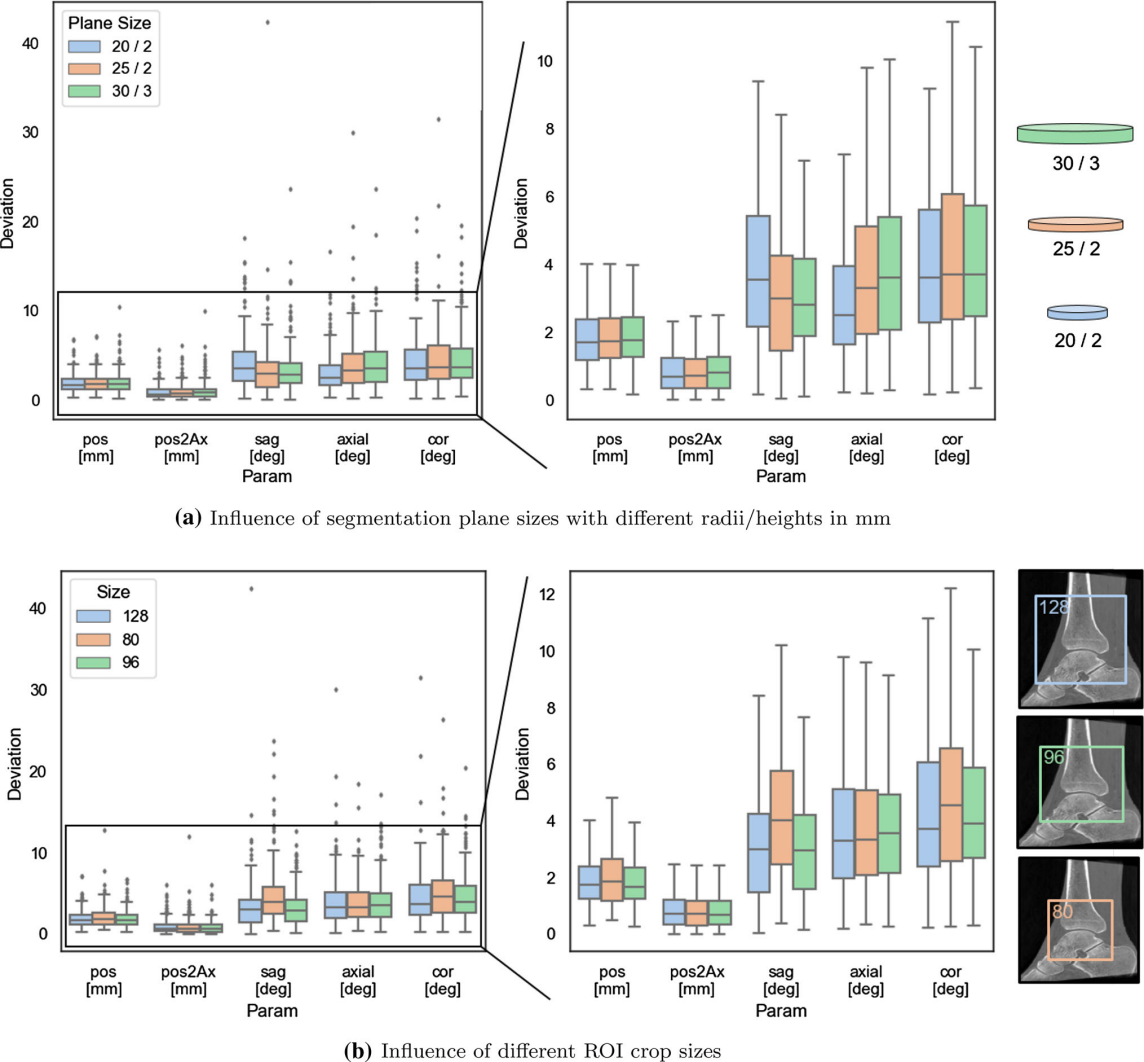
Plane estimation: Influence of image crop size and segmentation mask size. The position and angle deviation is evaluated on unilateral ankle data in a five-fold cross-validation. Both plots depict the error distribution and indicate that the results only change by a small margin under different setups

Figure 5 depicts the positional deviation between the ground truth center and the predicted center. The mean Euclidean distance is $4.01\pm 3.75$ mm with a maximum of 29.83 mm.

**Plane estimation:** Experiments were conducted to evaluate the standard plane estimation under different setups on the unilateral ankle data. The network was implemented with Pytorch and trained using a 12GB Geforce Titan X. The training cycle iterated over 300 epochs with a batch size of 4 image volumes.

The accuracy of the plane estimation was assessed in a five-fold cross validation by setting the crop size to 128 and the mask size radius to 25. Martin Vicario et al. [11] evaluated their plane estimation approach based on CNN regression on the same unilateral ankle data so that their results can be compared directly to the results of the proposed approach. In both approaches, the images were downsampled and normalized differently. However, the results in Table 1 indicate that plane estimation via segmentation decreases the median positional and angular error.

**Fig. 7:**
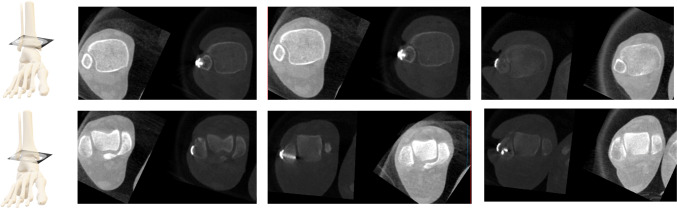
Overall system evaluation: Qualitative results of all 3 clinical bilateral ankle scans: The upper row shows the plane at 10 mm above the tibia plateau (tibiofibular incisura) and the lower row shows the planes 6 mm below the tibia plateau (talar dome level)

To assess the influence of the segmentation shape, the height and radius of the plane cylinder masks were varied. Smaller radii on the one hand can affect the robustness of the regression step. Larger radii on the contrary can lead to inaccuracies if the cropped ROI does not contain the entire cylinder. The mask radii were set to 20, 25 and 30 mm with heights of 2 and 3 mm. The ROI crop size was set to 128 voxel. The results shown in Fig. 6a do not reveal large differences between the different plane mask sizes. To examine the influence of the ROI size on the segmentation accuracy, different crop sizes were chosen. A smaller ROI naturally leads to less computation time. On the downside, the ROI has to be carefully chosen so that it contains all relevant information and is placed at the relevant location. For training, the ROI was sampled from an image subregion so that translation to the ideal iso-center could be augmented. Here, the cylinder mask radius was set to 25 mm. Figure 6b depicts results for crop sizes of 80, 96 and 128 voxel. Crop size 80 yielded slightly worse results for the sagittal and coronal normal deviation.

**Overall system evaluation:** In this experiment, the overall system accuracy was examined on 7 bilateral cases. Evaluation requires ground truth annotations of both ankles. The two-step approach was executed on each image volume. As a first step, the localization was computed by applying the network in a sliding window approach. As patch size $64^3$ was used with a stride of 32. Thereafter, the standard plane estimation was performed on each cropped subregion. A cropped voxel size of 80 was chosen for the experiments so that the cropped region did not contain parts of the second ankle. The pipeline outputs the intersection center as well as three normal vectors that can be compared to the ground truth. The same metrics as for the plane estimation of unilateral ankles were applied. The quantitative results in Table 1 show that the accuracy is similar to the accuracy achieved with unilateral ankle volumes but varies for the different specimens. For unilateral ankles, the experiment yielded a median deviation of $1.77\pm 1.48$ mm (pos), $0.72\pm 1.36$ mm (pos2Ax - position to axial plane), $2.98\pm 3.50^\circ$ (axial), $3.32\pm 5.17^\circ$ (sagittal), and $3.71\pm 5.13^\circ$ (coronal). The standard deviation was computed over all datasets and for comparison to the state of the art over the folds as displayed in Table 1. In Fig. 7, the visual outcome of the clinical cases is depicted on two different levels.

## Discussion

In this paper, a full pipeline for contralateral side comparison through standard plane adjustment was presented. The experiments showed that a segmentation approach yields accurate results on unilateral ankle data. Applying the pipeline to images with two ankles reaches similar accuracy even though the networks were solely trained on single ankles. The segmentation approach outperformed the regression method by Martin Vicario et al. [11] and seems to be more suitable for the pipeline. The translational discrepancy of that approach might be explained by the downsampling to $72^3$ voxels. For better comparison, the standard deviation of the proposed approach in the Table 1 is reported similar to the state of the art over the median folds instead of over all datasets. The standard deviation computed over all datasets is considerably higher which can be explained by looking at the distribution shown in Fig. 6a. The proposed network learns the cylinders’ shape and only changes the position and orientation. Even though the segmentation masks are entirely artificial and do not follow any distinct image features, the network can still learn the representation. This again underlines the power of the U-net. The largest outliers of the unilateral ankle experiment result from images with ankles located far from the isocenter. This is not extensively covered during training and aggravated by the center crop performed in testing. The system fails to recover a plane estimation. However, the localization step of the final application is targeted at placing the ankle close to the image volume center. The dataset acquired during the surgical course in particular contains complex and difficult fractures that are synthesized with a high amount of metal implants. This causes two challenges for the network: 1) There are large areas of metal artifacts, sometimes present at locations not included in the training. 2) Comminuted fractures may alter the entire shape representation of the ankle but are a crucial feature for the network to predict the right alignment. The manual alignment of those cases also has proven to be difficult. Therefore it seems natural that the performance on those corner cases is worse. Still, the network is capable to predict a quite close estimate as shown in Fig. 6.

For both steps, the same U-net architecture was used to examine whether the network is capable of segmenting artificially created shapes. The segmentation approach worked reasonably well for the coarse initialization step in the pipeline. The proposed approach is comparable to landmark regression with the difference that the landmark point is enlarged to represented a sphere instead of the more common definition of landmarks with Gaussian heatmaps. Preliminary experiments showed that the combination of binary segmentations with a combined cross-entropy and soft dice loss could handle the high class-imbalance of fore- and background better than heatmap approaches. Besides landmark regression methods, often object detection methods are used to estimate the region of interest. It is worth comparing these approaches to the patch-based initialization step in the future. With a sufficiently large amount of data, networks like the faster R-CNN [20] or one-stage Retina Net [19] may further improve the robustness of the initialization and increase the speed of the overall pipeline.

Even though a better accuracy was achieved compared to other approaches, the presented application is really sensitive to offsets that are directly noticeable by visual perception. The images are downsampled to a spacing of 1 mm due to the computational capacity, which naturally restricts the positional accuracy. Enlarging the amount of training images and at the same time the resolution might already have a positive affect on the outcome. Up to that point, the prototype offers a correction tool to handle positional offsets easily. In the future, inter-rater studies will help to further quantify the accuracy requirements, although the exact requirement is difficult to assess. The prototype could be directly integrated into clinical workflow, the main difference being the fixation of the second ankle joint so that both can be acquired simultaneously. For all bilateral cases in this preliminary study, it was possible to fit both ankles in the field of view. However, more studies are necessary to identify possible limitations when dealing with large patients. Additionally, a usability study will give more insights on how well the software tool is received and to what extent the users need to correct the results. The pipeline execution time varies with the used GPU and ranges from 5 to 15 s. The main bottleneck is caused by network initialization and image preprocessing and probably can be further optimized in the future.

The proposed approach is a two-step approach in which the second step crucially depends on the first. An alternative approach would have been to execute the plane estimation in a patch-based manner. However, the algorithm seems to benefit from the assumption that the outcome is shape consistent which is violated for smaller patches. We believe that once the acquisition of both ankles is integrated into clinical routine-leading to more and diverse training data-the pipeline can be further optimized. The performance of the pipeline is demonstrated for the ankle but could be easily transferred to other symmetric anatomies.

## Conclusion

The presented application allows a surgeon to directly look at relevant anatomical structures and offers a side-by-side visualization of both ankles. This is the first prototype towards intra-operative contralateral side comparison with only a single C-arm volume. The first proof of concept is provided that the technical pipeline is capable of solving the standard plane estimation task of two ankles even if the training data only consists of single ankles. Defining the task as segmentation problem with artificial segmentation masks can yield very good results and improve the plane estimation in terms of angle and position accuracy. Future work will include a clinical study to validate the benefits of acquiring bilateral scans. Overall, the application can potentially improve the ankle reduction evaluation process, avoid revision surgeries and bring long-term benefits for the patients.
